# Mapping of Preoperative Screening Tools Reveals Urgent Need for Standardization in Gastrointestinal Cancer Surgery: A Scoping Review

**DOI:** 10.1002/wjs.70313

**Published:** 2026-03-10

**Authors:** Alexandria Paige Petridis, Jack Reeves, Cherry Koh, Michael Solomon, Sascha Karunaratne, Kate Alexander, Nicholas Hirst, Neil Pillinger, Linda Denehy, Bernhard Riedel, Chelsia Gillis, Sharon Carey, Kate McBride, Kate White, Haryana M. Dhillon, Patrick Campbell, Raaj Kishore Biswas, Daniel Steffens

**Affiliations:** ^1^ Surgical Outcomes Research Centre (SOuRCe) Royal Prince Alfred Hospital Sydney Australia; ^2^ Faculty of Medicine and Health Central Clinical School The University of Sydney Sydney Australia; ^3^ Graduate School of Health Faculty of Health University of Technology Sydney Sydney Australia; ^4^ NHMRC Clinical Trials Centre The University of Sydney Sydney Australia; ^5^ Institute of Academic Surgery (IAS) Royal Prince Alfred Hospital Sydney Australia; ^6^ Department of Anaesthesia Perioperative Medicine, and Pain Medicine Peter MacCallum Cancer Centre Melbourne Australia; ^7^ Department of Health Services Research Allied Health Peter MacCallum Cancer Centre Melbourne Australia; ^8^ Department of Physiotherapy Faculty of Medicine Dentistry and Health Sciences The University of Melbourne Melbourne Australia; ^9^ The Sir Peter MacCallum Department of Oncology, and The Department of Critical Care The University of Melbourne Melbourne Australia; ^10^ School of Human Nutrition McGill University Montreal QC Canada; ^11^ Faculty of Science School of Psychology Psycho‐Oncology Cooperative Research Group The University of Sydney Sydney Australia; ^12^ Charles Perkins Centre School of Health Sciences Faculty of Medicine and Health The University of Sydney Sydney Australia

**Keywords:** gastrointestinal cancer, multidisciplinary, scoping review, screening tools

## Abstract

**Background:**

Gastrointestinal (GI) cancers are a major global health challenge due to their high incidence, mortality, and surgical complication rates. Preoperative physical, nutritional, and psychological vulnerabilities increase the risk of adverse surgical outcomes. Despite this, there is currently no validated, self‐report screening tool integrating assessment across all three domains. This scoping review aims to identify and describe existing preoperative screening tools used to assess modifiable physical, nutritional, and psychological domains in adult patients undergoing elective GI cancer surgery.

**Methods:**

We conducted this scoping review in accordance with Arksey and O'Malley's framework and PRISMA‐ScR guidelines. Searches were performed across MEDLINE, EMBASE, CINAHL, EBM, and PsycINFO date limited from January 2000 to March 2025. Studies were included if they evaluated preoperative screening tools for physical, nutritional, and/or psychological assessment in adult patients undergoing GI cancer surgery. Data on tool characteristics, domains assessed, administration time, and psychometric properties were extracted and synthesized descriptively.

**Results:**

From 2825 initial records, 121 studies were included, encompassing 77 unique screening tools. These were categorized as physical (*n* = 21), nutritional (*n* = 16), and psychological (*n* = 40) tools. Most tools were brief (1–15 items).

**Conclusions:**

Although most screening tools are brief, feasible for self‐administration, and freely accessible, none integrated all three domains. Substantial heterogeneity in tools highlights the need for a comprehensive, validated multidomain preoperative screening tool for this population.

## Graphical abstract

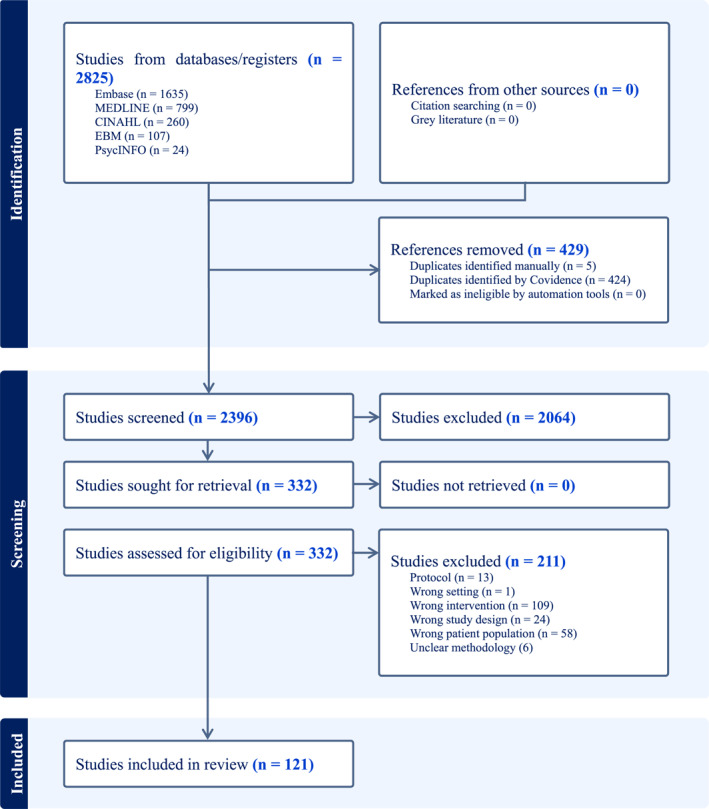

## 1 Introduction

Gastrointestinal (GI) cancers represent a significant global health burden due to their high incidence and mortality rates. According to global cancer statistics from 2022, colorectal cancer ranked 3rd in incidence and 2nd in mortality, pancreatic cancer 6th in cancer‐related mortality, and esophageal cancer 11th in incidence and 7th in mortality [1]. Collectively, GI cancers comprised 24.6% of all new cancer diagnoses and were responsible for 34.2% of global cancer‐related deaths [1]. It is estimated that total annual cancer incidence could increase by 77% by 2050 [1].

The primary curative modality for many GI cancers remains surgical intervention; however, this carries a high risk of postoperative complications reported in up to one third of patients [2]. This delays recovery, prolongs hospitalization, reduces quality of life, and increases healthcare costs [2, 3]. Poorer preoperative physical, nutritional, and psychological health are well‐documented predictors of adverse surgical outcomes [4, 5]. This highlights the importance of improved strategies in early detection, perioperative management, and treatment optimization to address the growing burden of GI malignancies.

Prehabilitation has emerged as a useful approach to increase physiological reserve prior to surgery and has been introduced to surgical oncology evolved to encompass physical, nutritional and psychological factors [6]. Despite the growing recognition that preoperative physical, nutritional, and psychological status influences postoperative outcomes, there remains no standardized, validated, and self‐administered screening tool to objectively measure all three domains. This scoping review synthesizes the available screening tools encompassing physical, nutritional, and/or psychological domains with a specific focus on their application in GI cancer patients and forms part of a broader research program investigating preoperative screening tools for people undergoing GI cancer surgery [7].

The aim of this scoping review is to identify and describe preoperative screening tools used in adults undergoing GI cancer surgery.

## 2 Methods

### 2.1 Study Design

This scoping review follows the methodological framework outlined by Arksey and O'Malley and will be reported in accordance with the PRISMA Extension for Scoping Reviews (PRISMA‐ScR) guidelines [8, 9]. The protocol for this review has been prospectively registered on the Open Science Framework (OSF) platform (https://doi.org/10.17605/OSF.IO/RMSB6).

### 2.2 Eligibility Criteria

Studies were included if they were peer‐reviewed reports in English published between January 2000 and March 2025 (to ensure tool use reflects recent practice), involved adult patients aged 18 years or older undergoing surgery for GI cancers (including colon, rectum, pancreas, stomach, liver, spleen, and esophagus), and assessed or screened for physical capacity/function, nutritional status, or psychological health in the preoperative period. Eligible study designs included randomized controlled trials, cohort studies, cross‐sectional studies, case series with > 10 participants and published conference abstracts.

Studies were excluded if they involved mixed surgical populations with > 80% noncancer patients, did not involve GI cancer surgery or focused on cancer types not specified above, or were case‐control studies or case studies/series with ≤ 10 participants.

### 2.3 Information Sources and Search Strategy

A comprehensive search was conducted on MEDLINE, EMBASE, evidence‐based medicine (EBM), Cumulative Index to Nursing and Allied Health literature (CINAHL) and PsycINFO electronic databases. Search strategies were developed in collaboration with a senior librarian from The University of Sydney. A combination of Medical Subject Headings (MeSH) and keywords were used, including but not limited to: “screening tools,” “gastrointestinal neoplasms,” “preoperative,” “nutrition,” “psychological,” and “exercise” (Table S1).

### 2.4 Gray Literature

In addition to database searches, a gray literature search was conducted to identify relevant tools and studies not captured in searched databases. A process of “pearling” was employed, whereby the reference lists of all included articles were examined to identify additional studies of interest. Forward citation tracking was performed using Google Scholar to capture more recent studies, which cite the included works. This strategy ensured a comprehensive search by identifying unpublished or informally published materials that may contribute valuable insights into existing preoperative screening tools used in GI cancer surgery [10].

### 2.5 Selection of Included Studies

All identified citations were imported into systematic review software (Covidence) for de‐duplication and subsequent screening [11]. Two independent reviewers (AP and JR) conducted title and abstract screening against the predetermined eligibility criteria. Subsequently, full‐text articles were retrieved and assessed for final inclusion. The study selection process was documented at each stage, including the number of articles included and excluded, along with rational for exclusion. Inter‐reviewer disagreements were resolved through discussion or escalation to a third reviewer (DS) when consensus could not be achieved.

### 2.6 Data Extraction, Charting Process, and Synthesis of Results

Data extraction was performed independently by two reviewers (AP and JR) using a standardized extraction template in Covidence [11]. For each included study, information on study and participant characteristics were recorded, including, author(s), title, publication year, study design, GI cancer subtype, and sample size. Screening tool attributes were also captured in detail, including tool name, year of original development, recall period, number of items, domains assessed, scoring metric, scoring range, estimated completion time, response format, and accessibility (i.e., free vs. licensed).

Extracted data were synthesized descriptively and screening tools categorized by domain (physical, nutritional, and psychological). Findings were then presented in tabular and narrative formats.

## 3 Results

### 3.1 Study Selection

**FIGURE 1:**
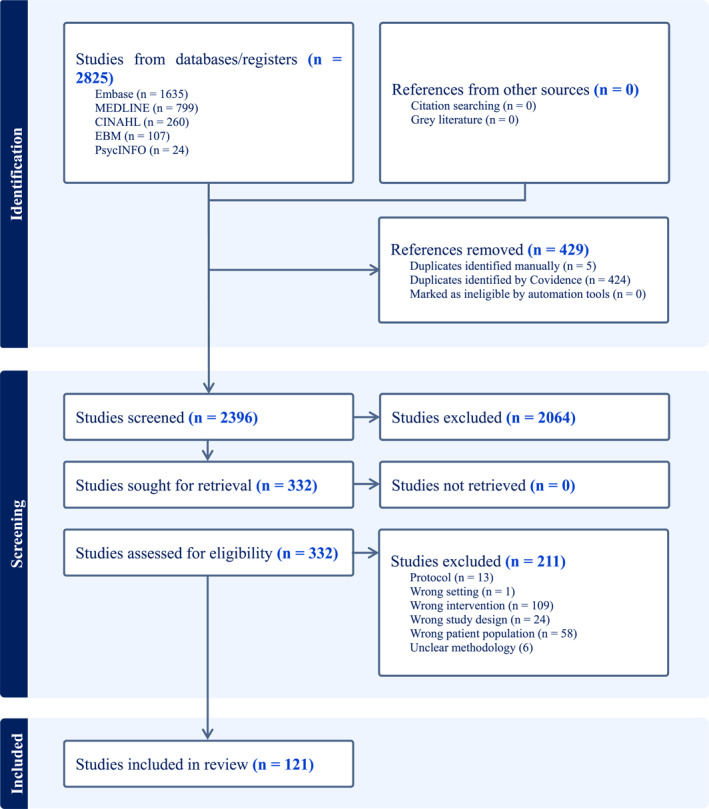
PRISMA diagram for a scoping review to identify and describe existing preoperative screening tools used to assess modifiable physical, nutritional, and psychological domains in adult patients undergoing elective gastrointestinal cancer surgery.

A total of 2825 records were identified through electronic database searches, including EMBASE (*n* = 1635), MEDLINE (*n* = 799), CINAHL (*n* = 260), evidence‐based medicine (*n* = 107), and PsycINFO (*n* = 24). After removal of 429 duplicates, 2396 records remained for title and abstract screening. A total of 332 full‐text articles were reviewed in detail, identifying 121 studies that met all eligibility criteria (see Figure 1 PRISMA diagram). Studies were excluded primarily due to: (i) not involving surgical populations; (ii) not using a screening tool preoperatively; or (iii) not including > 80% GI cancer patients (or lack of information regarding the specific patient population).

### 3.2 Characteristics of Included Studies

Characteristics of included studies can be seen in Table 1. Most studies were prospective cohort designs (*n* = 63, 52%) followed by retrospective cohort designs (*n* = 10, 8%). Geographically, studies originated from 27 countries with the highest representation of studies being from China (*n* = 24, 20%), the United Kingdom (*n* = 13, 11%), the United States of America (*n* = 9, 7%), and Sweden (*n* = 9, 7%). All screening tools were applied between 48 h and 6 weeks preoperatively. Sample sizes ranged from 17^12^ to 1687 participants [95] with a median sample size of 140 participants. Cancer populations included were predominantly colorectal (*n* = 64, 41%), gastric (*n* = 26 17%), esophageal (*n* = 14, 10%) and pancreatic (*n* = 12, 8%) either exclusively or part of a mixed GI cohort.

**TABLE 1:** Characteristics of studies included in the scoping review.

Author + year	Study design	Study duration	Questionnaires	Number of patients	Mean age (± SD or range)	Sex (%M:F)	Cancer type
Nutritional							
Agasi‐Idenburg 2020 [12]	Prospective exploratory study	2016–2018	Lawton Brody scale, multidimensional fatigue inventory‐short version (MFI‐20), short nutritional assessment questionnaire (SNAQ)	56	67 (median)	64:36	Colorectal
Al‐Bayyari 2024 [13]	Cross‐sectional	2018–2020	Malnutrition universal screening tool (MUST)	100	59.2 ± 9.8	60:40	Gastric, colorectal
Almasaudi 2019 [14]	Prospective cohort study	2013–2016	Malnutrition universal screening tool (MUST)	363	66 ± 12	55:45	Colorectal
Andrew 2020 [15]	Abstract—prospective cohort study	2019	Malnutrition screening tool (MST)	38	Not reported	Not reported	Gastrointestinal
Berstad 2013 [16]	Observational study	2006–2009	Self‐administered food‐frequency questionnaire (FFQ)	100	67	57:43	Colorectal
Budzynski 2024 [17]	Prospective cohort study	2016–2019	Barthel index [BI], lawton instrumental activities of daily living scale, mini nutritional assessment (MNA), nutritional risk screening (NRS‐2002), patient‐generated subjective global assessment (PG‐SGA)	84	66.7 ± 11.0 + 67.9 ± 12.2	66.7:33.3 + 52.4:47.6	Colorectal
Burden 2010 [18]	Prospective cohort study	N/A	Malnutrition universal screening tool (MUST)	87	64 (range 23–90 years)	62:38	Colorectal
Casirati 2022 [19]	Retrospective cohort study	2019–2020	Malnutrition universal screening tool (MUST)	100	61 (range 48.5–68)	48:52	Retroperitoneal sarcoma
Chen 2024 [20]	Randomized control trial	2019–2023	3‐Day total food recall questionnaire, hospital anxiety & depression scale (HADS), nutritional risk screening (NRS‐2002)	115	Intervention: 73, control: 74	Intervention: 57.9:42.1. Control 51.7:42.1	Colorectal
Chi 2017 [21]	Prospective cross‐sectional	2012	Nutritional risk screening (NRS‐2002)	280	62.9 ± 11.9	59.3:40.7	Gastrointestinal, colorectal
Chwesiuk 2023 [22]	Abstract—prospective cohort study	N/A	Nutritional risk screening (NRS‐2002), self‐administered food‐frequency questionnaire (FFQ), short nutritional assessment questionnaire (SNAQ), the strength assistance in walking rise from a chair climb stairs and falls (SARC‐F)	32	66.0 ± 9.7	59:41	Colorectal
Dias Rodrigues 2017 [23]	Prospective observational intervention study	2012–2014	Patient‐generated subjective global assessment (PG‐SGA)	37	60.2 ± 10	59.5:49.5	Gastric
Dou 2020 [24]	Prospective observational cohort study	2015–2016	Nutritional risk screening (NRS‐2002)	201	64.7 ± 10.5	74.6:25.4	Gastrointestinal
Driessens 2022 [25]	Prospective cohort study	2019–2021	Hospital anxiety & depression scale (HADS), patient‐generated subjective global assessment (PG‐SGA)	137	69.0 (63–74)	53.3:46.8	Hepato‐pancreato‐biliary (HPB)
Dubey 2024 [26]	Prospective cross‐sectional study	2018–2019	Malnutrition universal screening tool (MUST)	24	N/A	47:53	Hepato‐pancreato‐biliary (HPB)
Elsherbini 2024 [27]	Abstract—retrospective cohort study	2019–2020	Malnutrition screening tool (MST)	519	N/A	N/A	Upper gastrointestinal, lower gastrointestinal, thoracic
Fukuda 2016 [28]	Prospective cohort study	2012–2015	Food frequency questionnaire	99	Sarcopenic: 78 (R 67–85) non‐sarcopenic: 75 (R 66–91)	66.7:33.3	Gastric
Fulop 2021 [29]	Randomized control trial	2017–2019	Hospital anxiety & depression scale (HADS), malnutrition universal screening tool (MUST)	184	Intervention: 70 (IQR 60–75) control: 70 (IQR 64–75)	Intervention: 43:57 control: 54:47	Colorectal
Gillis 2015 [30]	Prospective observational study	2013	Patient‐generated subjective global assessment (PG‐SGA)	70	66.4 (± 12)	61:39	Colorectal
Gillis 2022 [31]	Pooled analysis	2013–2019	Hospital anxiety & depression scale (HADS), patient‐generated subjective global assessment (PG‐SGA), community healthy activities model program for seniors questionnaire (CHAMPS)	266	Intervention: 69.6 ± 11.3 control: 74.6 ± 10.8	57.9:42.1	Colorectal
Guinan 2018 [32]	Prospective observational cohort study	N/A	European prospective investigation of cancer food frequency questionnaire (EPIC FFQ), short nutritional assessment questionnaire (SNAQ)	28	62.9 ± 8.2	82:18	Esophageal
Guo 2010 [33]	Prospective cohort study	2004–2007	Nutritional risk screening (NRS‐2002)	314	60 (R 24–94)	67.4:32.6	Gastric
Heckler 2021 [34]	Prospective cohort study	2015–2017	Malnutrition universal screening tool (MUST), mini nutritional assessment (MNA), mini‐nutritional assessment‐short form (MNA‐SF), nutritional risk screening (NRS‐2002), nutritional risk screening score (NRS), short nutritional assessment questionnaire (SNAQ)	116	65 ± 11	46.6:53.4	Pancreatic
Hsueh 2020 [35]	Retrospective cohort study	2007–2014	Malnutrition universal screening tool (MUST), nutritional risk screening (NRS‐2002), patient‐generated subjective global assessment (PG‐SGA)	272	65.7 (R 26.3–97.2)	64.7:35.3	Gastric
Hua 2022 [36]	Prospective cohort study	2018–2019	Malnutrition universal screening tool (MUST), mini‐nutritional assessment‐short form (MNA‐SF), nutritional risk screening (NRS‐2002)	219	66	66.7:33.3	Esophageal
Huang 2020 [37]	Prospective cohort study	2014–2018	Malnutrition screening tool (MST), malnutrition universal screening tool (MUST), nutritional risk screening (NRS‐2002), short nutritional assessment questionnaire (SNAQ)	880	64.5 (± 10.8)	73.8:26.1	Gastric
Karanikki 2024 [38]	Prospective cross‐sectional observational study	2022–2023	Patient‐generated subjective global assessment (PG‐SGA)	480	CR: 69.2 ± 11.7. HPB 63.8 ± 12.4. Upper GI: 66.6 ± 11.2	CR: 62:38. HPB & upper GI: 67:33	Colorectal, hepato‐pancreato‐biliary (HPB), upper gastrointestinal
Karin 2020 [39]	Prospective observational study	2013–2015	Malnutrition universal screening tool (MUST), patient‐generated subjective global assessment (PG‐SGA)	127	67 ± 11	71:37	Colorectal
Karlsson 2009 [40]	Retrospective cohort study	2003–2005	Patient‐generated subjective global assessment (PG‐SGA)	153	70 (R 41–92)	50:50	Colorectal
Kim 2018 [41]	Abstract—prospective cohort study	2008–2014	Mini nutritional assessment (MNA)	154	N/A	N/A	Periampullary
Klassen 2020 [42]	Prospective cohort study	2016–2017	Patient‐generated subjective global assessment short form (PG‐SGA SF)	176	63.8 ± 12	52.3:47.7	Colorectal
Kollar 2022 [43]	Prospective non‐randomized interventional study	2016–2018	Nutritional risk screening (NRS‐2002)	259	68.2 ± 10.7	58.7:41.3	Colorectal
Kopuz 2023 [44]	Abstract—prospective observational study	N/A	Nutritional risk screening (NRS‐2002)	51	61.66 ± 11.9 years;	62.7:37.2	Colorectal
Lazaro 2023 [45]	Abstract—prospective observational study	2019–2020	Malnutrition universal screening tool (MUST)	Not reported	N/A	N/A	Colorectal
Lewicka 2019 [46]	Abstract—prospective control study	N/A	Nutritional risk screening (NRS‐2002)	143	N/A	N/A	Gastrointestinal
Lidoriki 2022 [47]	Prospective cohort study	2015–2019	Patient‐generated subjective global assessment (PG‐SGA)	98	60.79 ± 10.19	80.6:19.4	Gastroesophageal
Loh 2012 [48]	Prospective cohort study	2011	Malnutrition universal screening tool (MUST)	104	64.7 ± 10.8	60.6:39.4	Esophageal, gastric, pancreatic
Lu 2022 [49]	Prospective cohort study	2020	Mini sarcopenia risk assessment (MSRA‐5), mini sarcopenia risk assessment (MSRA‐7), nutritional risk screening (NRS‐2002), the strength assistance in walking rise from a chair climb stairs and falls (SARC‐F)	263	62.38 ± 11.21	75.4:24.6	Gastric
Lu 2022 [50]	Prospective cross‐sectional	2020	Nutritional risk screening (NRS‐2002), the strength assistance in walking rise from a chair climb stairs and falls (SARC‐F)	260	62.38 ± 11.21	75.4:24.6	Gastric
Niemelainen 2022 [51]	Multicentre observational study	2019–2020	Mini‐nutritional assessment‐short form (MNA‐SF), onco‐geriatric screening tool (G8)	167	84.5 (R 80–97)	59.3:40.7	Colon
Noh 2022 [52]	Retrospective cohort study	2012–2016	Nutritional risk screening (NRS‐2002)	274	63 (IQR 58–70)	94.5:5.5	Esophageal
Oikonomou 2024 [53]	Abstract—prospective cohort study	2022–2023	Duke activity status index (DASI), patient‐generated subjective global assessment (PG‐SGA)	50	Not reported	54:46	Hepato‐pancreato‐biliary (HPB)
Pellegrinelli 2024 [54]	Retrospective cohort study	2015–2022	Malnutrition universal screening tool (MUST)	79	G1: 63.1 ± 11.5. G2: 67.3 ± 8.5	G1: 50:50. G2: 60.7:39.3	Pancreatic
Reisinger 2015 [55]	Retrospective cohort study	2010–2012	Short nutritional assessment questionnaire (SNAQ)	210	69	50:50	Colorectal
Ryu 2010 [56]	Prospective cohort study	2005–2006	Nutritional risk screening (NRS‐2002)	80	G1: 58.5 ± 11.9	54:46	Gastric
					G2: 56.5 ± 13.2		
Tu 2012 [57]	Prospective cohort study	−2017–2019	Malnutrition universal screening tool (MUST)	45	62.1 ± 11.5	56:44	Colorectal
van der Kroft 2018 [58]	Prospective cohort study	2012–2013	Malnutrition universal screening tool (MUST)	63	69 ± 10.5	64:36	Colorectal
Van Wijk 2021 [59]	Prospective cohort study	2019–2021	Hospital anxiety & depression scale (HADS), patient‐generated subjective global assessment (PG‐SGA)	100	73 (IQR 66–76)	51:50	Hepato‐pancreato‐biliary (HPB)
Wang 2016 [60]	Prospective cohort study	2014–2015	Nutritional risk screening (NRS‐2002)	255	65.14 ± 10.81	74.5:25.5	Gastric
Wang 2018 [61]	Prospective cohort study	2016–2017	Nutritional risk screening (NRS‐2002)	60	N/A	90:10	Esophageal
Wang 2021 [62]	Prospective cohort study	2018–2019	Malnutrition universal screening tool (MUST), mini‐nutritional assessment‐short form (MNA‐SF), nutritional risk screening (NRS‐2002)	189	65.1 ± 7.2	68.8:31.2	Esophageal
Wiljma 2024 [63]	Prospective cohort study	2021–2023	Patient‐generated subjective global assessment (PG‐SGA)	30	G1: 60.5 (IQR 58–71.3)	15:85	Hepato‐pancreato‐biliary (HPB)
					G2: 70 (IQR 59.8–74.5)		
Wobith 2023 [64]	Retrospective cohort study	2017–2019	Nutritional risk screening (NRS‐2002)	260	N/A	N/A	Gastrointestinal
Yang 2022 [65]	Abstract ‐ prospective longitudinal study	N/A	Mini nutritional assessment (MNA)	112	N/A	N/A	Pancreatic
Yang 2024 [66]	Randomized control trial	2021	Hospital anxiety & depression scale (HADS), nutritional risk screening (NRS‐2002)	95	G1l 60 ± 10 G2: 64 ± 12	61:39	Colorectal
Yeung 2017 [67]	Prospective cohort study	2014–2015	Malnutrition screening tool (MST)	115	G1: 57 ± 13. G2: 61.6 14	58.3:41.7	Colorectal
Yong 2023 [68]	Prospective cohort study	2018–2022	Nutritional risk screening (NRS‐2002)	312	72.7	69.6:30.7	Gastric
Yoon 2018 [69]	Abstract—Prospective cohort study	2011–2012	Malnutrition screening tool for cancer patients (MSTC), malnutrition universal screening tool (MUST), nutritional risk screening (NRS‐2002), Seoul National University Bundang hospital nutritional screening tool (SNUBH‐NST)	170	N/A	N/A	Gastric, colorectal
Zhang 2022 [70]	Cross‐sectional study	2020	Mini‐nutritional assessment‐short form (MNA‐SF), nutritional risk screening (NRS‐2002)	265	70 (IQR 66–74)	72.8:27.2	Gastric, colorectal
Zhang 2023 [71]	Prospective cohort study	2021–2022	International physical activity questionnaire—short version (IPAQ‐SV)	359	G1: 63.90 ± 11.22 G2: 62.21 ± 11.17	64.3:35.7	Colorectal
Zhou 2025 [72]	Retrospective study	2019–2021	Nutritional risk screening (NRS‐2002)	145	64 (IQR 55–69.5)	73.8:26.2	Gastric, colorectal
Physical							
Ainsworth 2024 [73]	Abstract—prospective cohort study	2021–2022	International physical activity questionnaire‐short form (IPAQ‐SF)	50	63	58:42	Colorectal
Bae 2023 [74]	Abstract—retrospective cohort study	2019–2022	The strength assistance in walking rise from a chair climb stairs and falls (SARC‐F)	285	Not reported	Not reported	Colorectal
Cassini 2019 [75]	Abstract—prospective cohort study	2018–2019	Frailty index test questionnaire, the strength assistance in walking rise from a chair climb stairs and falls (SARC‐F)	24	74 ± 5.8	N/A	Colorectal
Chen 2017 [76]	Re‐analysis of preoperative data from two randomized control trials	N/A	Community healthy activities model program for seniors questionnaire (CHAMPS)	116	Intervention: 67.9 (± 1.5) control: 67.3 ± 1.2	63:37	Colorectal
Chwesiuk 2023 [22]	Abstract—prospective cohort study	N/A	Nutritional risk screening (NRS‐2002), self‐administered food‐frequency questionnaire (FFQ), short nutritional assessment questionnaire (SNAQ), the strength assistance in walking rise from a chair climb stairs and falls (SARC‐F)	32	66.0 ± 9.8	59:42	Colorectal
Dronkers 2010 [77]	Randomized control trial	N/A	LASA physical activity questionnaire	42	Intervention: 65.5 (± 6.4) control: 71.1 (± 6.3)	Intervention: 80:20 control: 68:32	Abdominal
Gillis 2022 [31]	Pooled analysis	2013–2019	Hospital anxiety & depression scale (HADS), patient‐generated subjective global assessment (PG‐SGA), community healthy activities model program for seniors questionnaire (CHAMPS)	266	Intervention: 69.6 (± 11.3) control: 74.6 (± 10.8)	57.9:42.2	Colorectal
Gray 2020 [78]	Observational cohort study	N/A	The patient reported outcome measurement information System‐10 (PROMIS‐10)	66	65 (± 12.5	49:51	Colorectal
Hernon 2021 [79]	Multicentre randomized control trial	2016–2018	Godin leisure time physical activity questionnaire	200	67.8 (R 35–86)	68:32	Colorectal, abdominal
Iwakura 2023 [80]	Prospective cohort study	2020–2021	International physical activity questionnaire‐short form (IPAQ‐SF), lowton instrumental activities of daily living scale, mini‐cog	97	74.4 ± 6.3	37.1:62.9	Gastric, colorectal, Gallbladder, bile duct, pancreatic
Karlsson 2019 [81]	Prospective observational cohort study	2015–2017	The physical activity scale for the elderly (PASE)	140	76 ± 4.6	62.9:37.1	Colorectal, bile duct, pancreatic
Kim 2025 [82]	Retrospective cohort study	2019–2022	The strength assistance in walking rise from a chair climb stairs and falls (SARC‐F)	285	High SARC‐F: 76.9 ± 8.5. Low SARC‐F: 64.5 ± 11.4	68.4:31.6	Colon
Komatsu 2018 [83]	Prospective cohort study	2013–2014	International physical activity questionnaire—short version (IPAQ‐SV), Kessler 6 (K6)	29	65.9 (R44.9–78.7)	93.1:6.9	Esophageal
Le Quang 2023 [84]	Re‐analysis of data from 6 RCTs and 1 cohort study	2011–2020	Community healthy activities model program for seniors questionnaire (CHAMPS)	459	G1: 72 (IQR 16). G2 (75 IQR 14)	56.6:43.4	Colorectal
Lin 2018 [85]	Prospective observational study	2013–2015	Hospital anxiety & depression scale (HADS), international physical activity questionnaire‐short form (IPAQ‐SF)	30	56.0 ± 15.2	53.3:46.7	Colorectal
Lu 2022 [49]	Prospective cohort study	2020	Mini sarcopenia risk assessment (MSRA‐5), mini sarcopenia risk assessment (MSRA‐7), nutritional risk screening (NRS‐2002), the strength assistance in walking rise from a chair climb stairs and falls (SARC‐F)	263	62.4 ± 11.2	75.4:24.7	Gastric
Lu 2022 [50]	Prospective cross‐sectional	2020	Nutritional risk screening (NRS‐2002), the strength assistance in walking rise from a chair climb stairs and falls (SARC‐F)	260	62.4 ± 11.2	75.4:24.7	Gastric
Meyers 2019 [86]	Prospective cohort study	2017–2018	Alcohol use disorder test‐clinician (AUDIT‐C), brief resillience scale, patient health questionnaire 2 (PHQ‐2), trauma survivors network recovery assessment Survey	143	65 (IQR 55–71)	55.9:44.1	Colorectal, hepato‐pancreato‐biliary (HPB)
Ngo‐Huang 2016 [87]	Abstract ‐ prospective observational study	N/A	PROMIS 12a Physical function short form	20	64	55:45	Pancreatic
Ngo‐Huang 2019 [88]	Prospective observational study	2015–2017	International physical activity questionnaire‐short form (IPAQ‐SF), PROMIS 12a Physical function short form	50	66 ± 8 years	52:48:00	Pancreatic
Oikonomou 2024 [53]	Abstract—prospective cohort study	2022–2023	Duke activity status index (DASI), patient‐generated subjective global assessment (PG‐SGA)	50	Not reported	54:47	Hepato‐pancreato‐biliary (HPB)
Olsson 2007 [89]	Prospective cohort study	N/A	Eating dysfunction scale (EDS), gastrointestinal symptom rating scale (GSRS)	24	Females: 60 (R 47–75). Males: 65 (R 51–83)	62.5:37.5	Gastric, pancreatic, GI cardiac, esophageal, bile duct
Onerup 2019 [90]	Prospective cohort study	2014–2015	Self‐reported four‐level Saltin‐Grimby physical activity scale	115	1: 74.5 (IQR 15). 2: 71 (IQR 11). 3: 67 (IQR 20)	55:45	Colorectal
Parker 2019 [91]	Mixed methods study	N/A	PROMIS 12a Physical function short form, physical activity readiness questionnaire (PAR‐Q), social support for exercise Survey (SSES)	50	66 ± 8	52:48	Pancreatic
Pecorelli 2016 [92]	Analysis of randomized control trials	2011–2014	Community healthy activities model program for seniors questionnaire (CHAMPS)	151	67 (IQR 65–69)	65:35	Colorectal
Pecorelli 2024 [93]	Prospective observational study	2020–2022	Duke activity status index (DASI), the patient reported outcome measurement information System‐10 (PROMIS‐10)	528	68 (IQR 59–75)	48.6:51.1	Pancreatic
PREPARE‐ABC Trial Collaborative 2021 [94]	Pilot randomized control trial	2016–2018	Godin leisure time physical activity questionnaire	200	IG1: 67.6 (35–86). IG2: 66.7 (39–84). IG3: 69.1 (53–85)	68:32	Colorectal
Reinwalds 2024 [95]	Prospective cohort study	2015–2019	Self‐reported four‐level Saltin‐Grimby physical activity scale	1687	71 ± 10	51.9:48.1	Colon
St‐Pierre 2022 [96]	Abstract ‐ retrospective study	2011–2020	Hospital anxiety & depression scale (HADS), community healthy activities model program for seniors questionnaire (CHAMPS)	459	M: 64. F: 68	56.6:43.5	Colorectal
van Zutphen 2017 [97]	Prospective cohort study	2010–2013	Short questionnaire to assess health enhancing physical activity (SQUASH)	515	65 ± 10	51:39	Colorectal
Yanagisawa 2022 [98]	Prospective cohort study	2016–2020	International physical activity questionnaire‐short form (IPAQ‐SF)	101	63 (IQR 63–77)	61.4:38.6	Gastrointestinal
Zhang 2013 [99]	Prospective cohort study	2008	Symptom checklist‐90 (SCL‐90)	60	59 ± 7	81.7:18.3	Esophageal
Zhang 2023 [71]	Prospective cohort study	2021–2022	International physical activity questionnaire—short version (IPAQ‐SV)	359	G1: 63.90 ± 11.22 G2: 62.21 ± 11.18	64.3:35.8	Colorectal
Psychological							
Acher 2020 [100]	Abstract—Prospective cohort study	N/A	National comprehensive cancer network distress thermometer	34	Not reported	Not reported	Gastrointestinal
Antoniadis 2024 [101]	Prospective cohort study	N/A	Hospital anxiety & depression scale (HADS), National comprehensive cancer network distress thermometer	118	70.5 ± 8.5	70:30	Colorectal
Baoyindeligeer 2020 [102]	Randomized control trial	N/A	The self‐rating depression scale (SDS), visual analog scale (VAS), the self‐rating anxiety scale (SAS)	130	55 ± 6.31	36:64	Esophageal
Bott 2025 [103]	Prospective non‐randomized trial	N/A	Shortened Warwick‐Edinburgh mental Well‐being scale (SWEMWBS)	41	Intervention: 63 + control: 65	Intervention: 81:19. Control 90:10	Esophageal
Calman 2021 [104]	Prospective longitudinal cohort study	5 Years	Centre of epidemiologic studies depression scale (CES‐D)	872	N/A	N/A	Colorectal
Chen 2024 [20]	Randomized control trial	2019–2023	3‐Day total food recall questionnaire, hospital anxiety & depression scale (HADS), nutritional risk screening (NRS‐2002)	115	Intervention: 73, control: 74	Intervention: 57.9:42.1. Control 51.7:42.1	Colorectal
Ding 2023 [105]	Prospective observational cohort study	2020–2021	Experiences in close relationship scale (ECR), family APGAR, frailty phenotype (FP), hospital anxiety & depression scale (HADS), international physical activity questionnaire‐short form (IPAQ‐SF), nutritional risk screening (NRS‐2002), social support rating scale (SSRS)	406	68.5 (IQR 65–73)	78.6:21.4	Gastric
Driessens 2022 [25]	Prospective cohort study	2019–2020	Hospital anxiety & depression scale (HADS), patient‐generated subjective global assessment (PG‐SGA)	137	69.0 (63–74)	53.3:46.7	Hepato‐pancreato‐biliary (HPB)
Foster 2016 [106]	Prospective cohort study	2010–2012	Centre of epidemiologic studies depression scale (CES‐D), medical outcome study (MOS), personal Wellbeing index‐audit (PWI‐A), positive & negative affect Schedule short form (PANAS), self‐efficacy for managing chronic disease scale, State‐trait anxiety inventory (STAI)	857	68.2 ± 10.7	59.6:40.4	Colorectal
Fujita 2003 [107]	Prospective cohort study	1999–2000	Hospital anxiety & depression scale (HADS)	36		61:39	Abdominal
Fulop 2021 [29]	Randomized control trial	2017–2019	Hospital anxiety & depression scale (HADS), malnutrition universal screening tool (MUST)	184	Intervention: 70 (IQR 60–75) control: 70 (IQR 64–75)	Intervention: 43:57 control: 54:46	Colorectal
Gillis 2022 [31]	Pooled analysis	2013–2019	Hospital anxiety & depression scale (HADS), patient‐generated subjective global assessment (PG‐SGA), community healthy activities model program for seniors questionnaire (CHAMPS)	266	Intervention: 69.6 (± 11.3) control: 74.6 (± 10.8)	57.9:42.3	Colorectal
Glass 2022 [108]	Quasi‐experimental study	N/A	Generalized anxiety Disorder‐7 (GAD‐7), National comprehensive cancer network distress thermometer	18	60 (R 40–79)	Not reported	Colorectal
Gonzalez‐Saenzde Tejada 2017 [109]	Multicentre cohort study	2010–2012	Barthel index [BI], Duke‐UNC functional social support questionnaire [FSSQ], hospital anxiety & depression scale (HADS)	972	67.5 (10.4)	62.3:37.8	Colorectal
Hao Law 2022 [110]	Prospective cohort study	2018–2021	Hospital anxiety & depression scale (HADS)	78	66 (R 45–89)	56.4:43.6	Colorectal
Hong 2015 [111]	Multicentre prospective cohort study	N/A	Cancer coping modes questionnaire (CCMQ), National comprehensive cancer network distress thermometer	165	62.2 (± 8.8)	77:33	Gastric
Iwakura 2024 [80]	Prospective cohort study	2020–2021	International physical activity questionnaire‐short form (IPAQ‐SF), lowton instrumental activities of daily living scale, mini‐cog	97	74.4 ± 6.4	37.1:62.10	Gastric, colorectal, gallbladder, bile duct, pancreatic
Jakobsson 2016 [112]	Prospective cohort study	2011–2013	State‐trait anxiety inventory (STAI)	105	68.5	49.5:50.5	Colorectal
Komatsu 2018 [83]	Prospective cohort study	2013–2015	International physical activity questionnaire—short version (IPAQ‐SV), Kessler 6 (K6)	29	65.9 (R44.9–78.7)	93.1:6.10	Esophageal
Law 2023 [113]	Prospective cohort study	2018–2021	Hospital anxiety & depression scale (HADS)	78	66 (R45–89)	56.4:43.6	Colorectal
Li 2021 [114]	Abstract—prospective cohort study	2018–2019	Hospital anxiety & depression scale (HADS)	70	N/A	N/A	Colorectal
Liedman 2001 [115]	Prospective cohort study	1984–1991	Body symptom scale (BSS), gastric Symptom rating scale (GSRS), Karnofsky performance status scale (KPS), mood adjective check list (MACL), Sickness impact profile (SIP)	33	66 (R 41–82)	65.6:34.4	Gastrointestinal
Lin 2018 [85]	Prospective observational	2013–2015	Hospital anxiety & depression scale (HADS), international physical activity questionnaire‐short form (IPAQ‐SF)	30	56.0 ± 15.3	53.3:46.8	Colorectal
Liu 2021 [116]	Prospective cohort study	2019–2020	Hospital anxiety & depression scale (HADS), surgical fear questionnaire (SFQ)	282	65.8 ± 12.0	61:39	Colorectal, gastric
Masui 2010 [117]	Prospective cohort study	2008	Hospital anxiety & depression scale (HADS)	17	63 ± 5.7	88:12	Esophageal
Mei 2022 [118]	Prospective cohort study	2015–2019	Hospital anxiety & depression scale (HADS)	125	1. 64.73 ± 8.83 Group 2. 63.37 ± 8.81	51.5:48.5	Rectal
Mesquita Garcia 2018 [119]	Randomized control trial	N/A	State‐trait anxiety inventory (STAI)	50	IG: 58 ± 11, CG: 57 ± 15	44:56	Colorectal
Olsson 2007 [89]	Prospective cohort study	N/A	Eating dysfunction scale (EDS), gastrointestinal symptom rating scale (GSRS)	24	Females: 60 (R 47–75). Males: 65 (R 51–83)	62.5:37.4	
Ono 2021 [120]	Prospective cohort study	2015–2020	Social frailty questionnaire, onco‐geriatric screening tool (G8), 15‐item geriatric depression scale	181	72	76.8:23.2	Gastric, esophageal, colorectal
Penning 2022 [121]	Retrospective study	2016–2019	Onco‐geriatric screening tool (G8)	112	74 (70–92)	41.1:58.9	Colorectal, gastric, Small bowel, esophageal
Piraux 2020 [122]	Prospective cohort study	2018–2019	Hospital anxiety & depression scale (HADS)	23	61.7 ± 10.6	69.6:30.4	Esophageal, gastric
Sarenmalm 2018 [123]	Prospective cohort study	N/A	National comprehensive cancer network distress thermometer, problem list	488	68 (R 32–93)	45:55	Colorectal
Sawatzky 2023 [124]	Prospective longitudinal study	2012–2015	Longitudinal preparedness for colorectal cancer surgery questionnaire (PCSQ), national comprehensive cancer network distress thermometer, sense of coherence short‐version scale	488	68 ± 11	56:44	Colorectal
Sharma 2007 [125]	Prospective cohort study	2003	Hospital anxiety & depression scale (HADS), positive & negative affect schedule short form (PANAS), the mood rating scale (MRS)	104	67.6 ± 10.4	67.3: 32.7	Rectal
Sharma 2013 [126]	Prospective cohort study	2003	Hospital anxiety & depression scale (HADS), positive & negative affect schedule short form (PANAS), the mood rating scale (MRS)	97	70 (39–86)	67:33	Rectal
Soler‐Silva 2022 [127]	Abstract ‐ randomized control trial	N/A	Hospital anxiety & depression scale (HADS)	25	N/A	N/A	Colorectal
St‐Pierre 2022 [96]	Abstract ‐ retrospective study	2011–2020	Hospital anxiety & depression scale (HADS), community healthy activities model program for seniors questionnaire (CHAMPS)	459	M: 64. F: 67	56.6:43.4	Colorectal
Sun 2020 [128]	Cross‐sectional study	2013–2017	10‐Item rosenberg self‐esteem scale	434	62.6 ± 11	58.5:41.5	Colorectal
Turner 2019 [129]	Prospective cohort study	N/A	Centre of epidemiologic studies depression scale (CES‐D)	857	N/A	N/A	Colorectal
Van Wijk 2021 [59]	Prospective cohort study	2019–2020	Hospital anxiety & depression scale (HADS), patient‐generated subjective global assessment (PG‐SGA)	100	72 (IQR 66–76)	51:49	Hepato‐pancreato‐biliary (HPB)
Xu 2016 [130]	Cross‐sectional study	2014–2015	Hospital anxiety & depression scale (HADS), medical coping modes questionnaire (MCMQ), social support rating scale (SSRS), the type D scale‐14 (DS‐14)	53	G1: 59.0 ± 10.4 G2: 58.1 ± 10.9	66:44	Gastric
Xu 2021 [131]	Prospective cohort study	2018	Brief illness perception questionnaire (BIPQ), hospital anxiety & depression scale (HADS), identity‐consequence fatigue scale (ICFS)	463	G1: 60.45 ± 10.35. G2: 56.16 ± 11.72	55.7:44.3	Esophageal, gastric, colorectal
Yang 2024 [66]	Randomized control trial	2021–2022	Hospital anxiety & depression scale (HADS), nutritional risk screening (NRS‐2002)	95	G1l 60 ± 10 G2: 64 ± 13	61:40	Colorectal

### 3.3 Characteristics of Identified Tools

Characteristics of tools identified can be seen in Table 2. A total of 77 unique screening tools were found. Most tools were concise with 71 (92%) of tools contained ≤ 15 items and requiring ≤ 10 min to complete. No self‐administered questionnaires that simultaneously assessed physical, nutritional, and psychological domains were found.

**TABLE 2:** Characteristics of screening tools identified in the scoping review.

Tool name	Times identified in search	Year developed	Recall period	Number of items	Response format	Score range	Time to complete	Tool availability	Total number of patients
Nutritional									
3‐Day total food recall	1	1947	3‐days	Variable	Open‐ended, detailed food diary	N/A	Variable (∼ 30–60 min)	Freely available	115
Alcohol use disorder identification test (AUDIT)	1	1989	12‐months	10	Likert scale	0–40	< 5 min	Freely available	143
Eating dysfunction scale (EDS)	1	2005	Past week	25	Likert scale	Variable	10 min	Restricted	24
European prospective investigation of cancer food frequency questionnaire (EPIC FFQ)	1	1989	Past year	130+	Multiple choice	N/A	30–60 min	Freely available for research use	28
Malnutrition screening tool (MST)	5	1999	6‐months	2	Yes/No	0–5	< 5 min	Freely available	1722
Malnutrition screening tool for cancer patients (MSTC)	1	2011	Current	4	Yes/No	0–4	< 5 min	Freely available	170
Malnutrition universal screening tool (MUST)	18	2003	Current	3	Scored components	0–2	< 5 min	Freely available	3122
Mini nutritional assessment (MNA)	4	1994	Current	18	Yes/No + scored items	0–30	10–15 min	Freely available for clinical use	466
Mini nutritional assessment short form (MNA‐SF)	5	2001	Current	6	Yes/No + scored items	0–14	10–15 min	Freely available for clinical use	956
Nutritional risk screening (NRS‐2002)	26	2002	Current	4	Scored items + disease Severity	0–7	< 5 min	Freely available	5660
Patient‐generated subjective global assessment (PG‐SGA)	12	2005	4‐week	4 Sections	Mixed	0–35+	10–15 min	Freely available for clinical use	1874
Patient‐generated subjective global assessment short form (PG‐SGA SF)/abridged PG‐SGA	2	2015	Current	4 components	Self‐reported checklist	0–36	5–10 min	Freely available	206
Self‐administered food‐frequency questionnaire (FFQ)	3	1970s–1980s	Past year (typically)	Varies widely (∼60–150 items)	Multiple choice	Not typically scored	20–60 min	Freely available	160
Seoul National University Bundang Hospital nutritional screening tool (SNUBH‐NST)	1	2012	Current	4	Checklist + score	0–8	< 5 min	Freely available in publications	170
Short/simplified nutritional assessment questionnaire (SNAQ)	6	2005	Past month	3 or 4	Yes/No	0–3 or 0–4	< 5 min	Freely available	1322
Physical									
Barthel index (BI)	2	1965	Current	10	Yes/No or scale	0–100	5 min	Freely available	2028
Body Symptom scale	1	N/A	1‐week	28 item or 17‐item short version	Likert scale	Variable	5–10 min	Restricted access	33
Community healthy activities model program for seniors questionnaire (CHAMPS)	5	2001	4‐week	41	Yes/No, frequency + duration	MET‐min/week	10‐20 min	Freely available	267
Duke activity status index (DASI)	2	1989	Current	12	Yes/No	0–58.2 or MET‐min/Week	5 min	Freely available	578
Gastric Symptom rating scale (GSRS)	1	1988	1‐week	15	Likert scale	1–7 per item (means score used)	5 min	Freely available for research use	33
Gastrointestinal Symptom rating scale (GSRS)	2	1988	1‐week	15	Likert scale	1–7 per item (means score used)	5 min	Freely available for research use	57
Godin leisure time physical activity questionnaire	2	1985	Typical week	4	Open‐ended frequency	Godin index score	5 min	Freely available	400
Groningen frailty indicator	3	1999	Current	15	Yes/No	0–15	5 min	Freely available	612
International physical activity questionnaire ‐ short version (IPAQ‐SV)	2	2001	1‐week	7	Open‐ended frequency + duration	MET‐min/week	5–10 min	Freely available	388
International physical activity questionnaire‐short form (IPAQ‐SF)	7	2001	1‐week	7	Open‐ended frequency + duration	MET‐min/week	5–10 min	Freely available	684
Karnofsky performance status scale (KPS)	1	2002	Current	1	Ordinal (increments of 10)	0–100	< 5 min	Freely available	33
LASA physical activity questionnaire (LAPAQ)	1	1991	2‐week	7	Likert scale	Variable	5–10 min	Freely available	42
Lawton Brody Scale/Lawton instrumental activities of daily living scale	3	1969	Current	8	3‐point scale (0,1,2)	0–8 or 0–16	5–10 min	Freely available	231
Mini sarcopenia risk assessment (MSRA‐5)	1	2017	1‐year	5	Yes/No	0–50	< 5 min	Freely available	263
Mini sarcopenia risk assessment (MSRA‐7)	1	2017	1‐year	7	Yes/No	0–70	< 5 min	Freely available	263
Patient reported outcome measurement information System‐10 (PROMIS‐10)	2	2009	Current	10	Likert scale	Raw score to T‐Score	< 5 min	Freely available from PROMIS initiative	593
Patient reported outcome measurement information System‐12a (PROMIS‐12a)	4	2010	1‐week	12	Likert scale	T‐score metric	< 5 min	Freely available with registration	120
Physical activity readiness questionnaire (PAR‐Q)	1	1978	Current	7	Yes/No	Binary clearance?	< 5 min	Freely available	50
Physical activity scale for the elderly (PASE)	1	1993	1‐week	12	Frequency + duration (weighted scoring)	0–309	10–15 min	Licensed (health assessment lab)	140
Self‐reported four‐level Saltin‐Grimby physical activity scale	2	1968 (revised 1990s)	Current	1	4 response levels	1–4	< 5 min	Freely available	1802
Short questionnaire to assess health enhancing physical activity (SQUASH)	1	2003	Typical week	∼13	Open‐ended + frequency/duration	MET‐min/week	5–10 min	Freely available	515
Strength assistance in walking rise from a chair climb stairs and falls (SARC‐F)	6	2013	Current	5	Likert scale	0–10	5 min	Freely available	1149
Psychological									
10‐Item rosenberg self‐esteem scale (RSES)	1	1965	Current	10	Likert scale	0–30	< 5 min	Freely available	434
15‐Item geriatric depression scale	1	1982	1‐week	15	Yes/No	0–15	5 min	Freely available	181
Brief illness perception questionnaire (brief IPQ)	1	2006	Current	9	Likert scale a+ 1 open‐ended item	0–80	5 min	Freely available	463
Brief resilience scale (BRS)	1	2008	Current	6	Likert scale	06–30 (Sum) or 1–5 (average)	5 min	Freely available	143
Cancer coping modes questionnaire	1	2003	Current	21	Likert scale		5–10 min	Restricted/academic use	165
Centre of epidemiologic studies depression scale (CES‐D)	3	1977	Past week	20	Likert scale	0–60	5–10 min	Freely available	2586
Duke‐UNC functional social support questionnaire [FSSQ]	1	1988	Current	11 or 8‐item short version	Likert scale	11‐Item: 11–55 or 8‐item: 8–40	< 5 min	Freely available	1944
Experiences in close relationship scale (ECR)	1	1998	Current	12	Likert scale	6–42	10 min	Freely available for academic use	406
Generalized anxiety Disorder‐7 (GAD‐7)	1	2006	2‐weeks	7	Likert scale	0–21	5 min	Freely available	18
Hospital anxiety & depression scale (HADS)	24	1983	1‐week	14	Likert scale	0–21 per subscale	5–10 min	Freely available for academic use	4333
Identity‐consequence fatigue scale (ICFS)	1	2006	1‐week	31	Likert	Variable	10–15 min	Academic use	463
Kessler psychological distress scale (K6)	1	2002	1‐month	6	Likert scale + frequency/duration	0–24	< 5 min	Freely available	29
Longitudinal preparedness for colorectal cancer surgery questionnaire (PCSQ)	1	2016	Current	28, 24, 14	Likert scale	28 item: 0–112. 24 item: 0–96. 14 item: 0–56	5–10 min	Research use only	488
Medical coping modes questionnaire (MCMQ)	1	1992	Current	20	Likert scale	20–80	5–10 min	Freely available for academic use	53
Mood rating scale (MRS)	2	1989	Current	6	Visual analog	0–100 per item	< 5 min	Freely available for research use	201
Multidimensional fatigue inventory‐short version (MFI‐20)	1	1995	3‐days	20	Likert scale	20–100	10 min	Freely available for academic use	56
National comprehensive cancer network distress thermometer	6	1998	Current	1 ± problem list	0–10 scale	0–10	< 5 min	Freely available	1311
Onco‐geriatric screening tool (G8)	3	2012	3‐months	8	Scored items + disease severity	0–17	< 5 min	Freely available	460
Patient health questionnaire 2 (PHQ‐2)	1	1999	2‐weeks	2	Likert scale	0–6	< 5 min	Freely available	143
Personal wellbeing index‐audit (PWI‐A)	1	2001	Current	9	Likert scale	0–100	< 5 min	Freely available with perission	857
Positive & negative affect schedule short form (PANAS)	3	1988	Past week	20	Likert scale	20–100	5 min	Freely available	1058
Problem list	1	2001	Current	∼34 items	Checklist	Not scored alone	< 5 min	Used with NCCN distress thermometer	488
Psychological distress inventory (PDI)	1	2003	1‐month	10	Likert scale	10–50	5–10 min	Freely available for research use	406
Rotterdam symptom checklist (RSCL)	1	1985	Past week	30	Likert scale	7–28 (Sum of 7 items)	10–15 min	Freely available for research use	990
Self‐efficacy for managing chronic disease scale	1	2003	Current	6	Likert scale	06–60	< 5 min	Freely available for non‐commercial use	857
Self‐rating anxiety scale (SAS)	1	1971	Past week	20	Likert scale	20–80	< 5 min	Freely available	130
Self‐rating depression scale (SDS)	1	1965	Past week	20	Likert scale	20–80	5 min	Licensed	130
Sense of coherence short‐version scale	1	1991	Current	13	Likert scale	13–91	5–10 min	Freely available for academic use	488
Shortened Warwick‐Edinburgh mental Well‐being scale (SWEMWBS)	1	2008	2012	7 (of original 13)	Likert scale	7–35	5–10 min	Licensed through Warwick innovations	41
Sickness impact profile (SIP)	1	1970s	Current	136 (short form: 68)	Yes/No	0–100	20–30 min	Licensed	33
Social frailty questionnaire	1	2017	Current	5–9 items	Yes/No or likert	Varies	5 min	Freely available	181
Social support rating scale (SSRS)	2	1982	Current	10	3 subscales, likert/quantitative	12–66	5–10 min	Freely available	459
State‐trait anxiety inventory (STAI)	3	1964	Current	20	Likert scale	20–80	10–15 min	Licensed (mind Garden inc.)	1012
Symptom checklist‐90 (SCL‐90)	1	1973	1‐week	90	Likert scale	0–360 OR 0–90	10–15 min	Licensed	60
The type D scale‐14 (DS‐14)	1	2005	Current	14	Likert scale	0–56	5–10 min	Freely available for research	53
Visual analog scale (VAS)	1	1921	Current	1	Numerical/scale	0–10	< 5 min	Freely available	130

### 3.4 Nutritional Screening Tools

There were 16 (21%) nutritional screening tools identified. Nutritional screening tools were often brief (1–18 items). However, the more comprehensive screening tools, such as food diaries (30–60 items), required longer to complete.

### 3.5 Physical Screening Tools

There were 21 (27%) physical screening tools identified. Physical function tools assessed patients' mobility, strength, endurance, and functional independence. These tools varied in administration method and completion time.

### 3.6 Psychological Screening Tools

Psychological tools made up the largest proportion of single domain tools with 40 (52%) tools identified. Psychological screening tools captured anxiety, depression, resilience, coping, and broader mental health parameters. Anxiety and depression tools were the most common psychological measures with the Hospital Anxiety and Depression Scale (HADS) appearing in 24 studies (60% of psychological domain). However, these tools were rarely the focus of the study and were included in studies evaluating quality of life.

### 3.7 Format, Administration, and Psychometrics

Screening tools exhibited substantial heterogeneity with respect to development period, format, and administration parameters. Although their initial publication dates span from 1921 (Visual analog scale; VAS) to 2017 (Mini‐Sarcopenia Risk Assessment; MSRA), the majority were introduced between the 1980s and early 2000s. Many have subsequently undergone modification often to create abbreviated versions or to facilitate patient self‐administration (e.g., the Patient‐Generated Subjective Global Assessment short form; PG‐SGA‐SF).

Scoring approaches varied with most tools generating either a continuous numeric score or categorical risk classification, whereas several incorporate domain‐specific subscale scoring. Completion times range from under 5 minutes for concise tools to 60 min for more elaborate assessments. With respect to accessibility, many tools are available without charge; however, a subset required formal licensing agreements or academic permissions. Item counts also demonstrated considerable variability from single‐item scales (e.g., visual analog scale (VAS); National Comprehensive Cancer Network Distress Thermometer; NCCN DT) to extensive questionnaires exceeding one hundred items (e.g., European Prospective Investigation of Cancer food frequency questionnaire; EPIC FFQ).

Notably, only 17 (14%) studies integrated assessments across multiple (i.e., at least two) domains (physical, nutritional, and psychological). Most tools (*n* = 71, 92%) comprised ≤ 15 questions and required less than 10 minutes to complete. Recall periods varied widely from “point‐in‐time” assessments (e.g., the Malnutrition Universal Screening Tool) to extended dietary recalls of up to 12 months (e.g., European Prospective Investigation of Cancer food frequency questionnaire; EPIC FFQ). However, most tools used a one‐week (46%) or one‐month (28%) recall period. Response formats were predominantly Likert scales (63%) or dichotomous yes/no items (21%) with open‐ended diary formats constituting fewer than 5% of tools. Accessibility was high with 84% of screening tools freely available and 16% requiring licensing or restricted permission.

## 4 Discussion

This review identifies 77 unique tools from 121 studies. These tools evaluated physical (*n* = 21), nutritional (*n* = 16), and psychological (*n* = 40) domains. Studies originated from 27 countries and were mostly prospective observational cohort studies by design (*n* = 63, 52%). Although most tools were brief, feasible to self‐administer, and freely accessible, none integrated all three domains within a single tool.

This review demonstrated a wide array of tools but an absence of validated, multidomain, and self‐reported tools for GI cancer surgery. Multidomain screening tools offer several advantages over single‐domain tools. By consolidating assessment across multiple domains into a single screening encounter, these tools may reduce the time and resource burden for both clinicians and patients. Rather than requiring multiple discipline‐specific assessments to be arranged preoperatively, multidomain tools can enable a single point of contact with targeted, discipline‐specific follow‐up initiated only when indicated by screening results. Furthermore, embedding a multidomain screening tool within a hospital model of care facilitates streamlined referral pathways, thereby improving workflow efficiency and optimizing use of healthcare resources. Existing single‐domain tools are typically short, feasible for self‐administration, and often freely available, yet heterogeneously applied and infrequently linked to standardized referral pathways or targeted prehabilitation programs or services.

The predominance of single‐domain screening may reflect the historical separation of perioperative assessment streams. Prehabilitation has matured largely within physical conditioning paradigms with nutrition and psychological care increasingly recognized but unevenly integrated into preoperative pathways [132]. The lack of psychological screening is noteworthy given the well‐described relationships between distress, mood disorders, treatment adherence, and postoperative recovery trajectories [109, 133]. Under‐recognition of psychological distress may adversely affect patients' engagement with prehabilitation and perioperative care contributing to poorer treatment adherence and recovery outcomes [134, 135]. Psychological distress can also impair information processing and participation in shared decision‐making potentially influencing treatment choices and expectations of surgical risk and recovery. Systematic psychological screening may therefore enable earlier identification and targeted referral, mitigating downstream impacts on perioperative outcomes. Similarly, nutritional risk remains highly prevalent in GI cancer and is modifiable, yet screening and referral is not uniformly embedded into models of care [18].

Several clinical implications arise from this study. First, clinicians involved in gastrointestinal cancer surgery should be aware of the fragmented approach to preoperative screening across physical, nutritional, and psychological domains. Although no validated multidomain screening tools comprehensively assess all domains in this population, surgeons, anesthetists, and allied health professionals should ensure that each domain is considered within preoperative assessment pathways given their independent associations with postoperative outcomes. This scoping review may assist multidisciplinary teams in selecting screening tools suited to their local clinical context, considering factors such as tool availability, recall period, time to complete, and response format (Table 2), especially important as prehabilitation is variably implemented as a standard of care and institutions face unique barriers globally [136]. In the absence of prospective evidence supporting specific multidomain tools, flexible and pragmatic screening strategies embedded within surgical models of care are warranted allowing tool selection and referral pathways to evolve as new evidence emerges.

This scoping review comprehensively mapped preoperative screening tools used in adult GI cancer surgery with a pragmatic focus on feasibility and real‐world application. The use of a comprehensive, multi‐database search and inclusion of multiple domains is a strength. However, heterogeneity in outcomes and populations (tumor sites, staging, neoadjuvant therapy), timing of assessment, and outcome definitions prevented pooled analysis and complicates direct comparisons across tools.

Restricting inclusion to English‐language publications and limited timeframes (2000–2025) may have missed tools in languages other than English or those published earlier, although this is unlikely as our search strategy was designed to capture tools used in recent practice (21^st^ century) even if developed previously. Additionally, this study aimed to identify and describe screening tools rather than evaluate tools based on their clinimetric properties. To ensure screening tools effectively identify those at risk, they need to be prospectively tested in the population and setting of interest (i.e., gastrointestinal cancer surgery). To address this limitation, the next phase of this research program involves development of a self‐reported, online screening tool to be assessed through an international cohort study based on findings from this scoping review.

Future studies should validate screening tools to gastrointestinal cancer surgery populations and assess responsiveness and minimal clinically important differences so that clinicians can interpret change scores during prehabilitation. Future work should also examine whether combining domain scores meaningfully improves risk stratification beyond single domains and whether the addition of patient‐reported outcomes enhances the performance of existing clinical risk models. Although multidomain screening tools may improve efficiency by consolidating assessment, the relative contribution of individual domains for identifying high‐risk patients undergoing gastrointestinal cancer surgery remains unclear. This scoping review was not designed to determine the comparative value or weighting of specific screening domains.

In addition, future prospective studies should seek to clarify the relative importance of individual screening domains and identify the minimum essential components required to balance clinical utility with feasibility in routine practice. Consideration should also be given to local service capacity, as longer and more complex tools may require additional time, staffing, digital infrastructure, and patient support, potentially limiting uptake in resource‐constrained settings. Tailoring tool length and mode of administration to center capabilities may therefore be critical for successful implementation.

Implementation research is required to determine how best to integrate screening into surgical pathways, including workflow mapping, role delineation, electronic health record integration, and automated prompts that convert scores to referrals. Given the widespread use of neoadjuvant therapy in GI cancer, studies should determine optimal timing and frequency of screening across treatment and the preoperative period to ensure evolving risks are identified and addressed.

## 5 Conclusions

This scoping review identified and mapped 77 self‐reported preoperative screening tools spanning physical, nutritional, and psychological domains in adult GI cancer surgical patients. Although most tools were brief, feasible to self‐administer, and freely accessible, none integrated all three domains within a single tool. The absence of a multidomain, psychometrically robust screening tool represents a clear gap. Future research could prioritize the development, validation, and implementation of a concise, multidomain tool to support personalized prehabilitation pathways and optimize surgical outcomes.

## Author Contributions

**Alexandria Paige Petridis:** conceptualization, methodology, formal analysis, data curation, project administration, writing – original draft, writing – review and editing. **Jack Reeves:** conceptualization, methodology, formal analysis, data curation, project administration, writing – review and editing. **Cherry Koh:** conceptualization, methodology, writing – review and editing. **Michael Solomon:** conceptualization, methodology, writing – review and editing. **Sascha Karunaratne:** conceptualization, methodology, writing – review and editing. **Kate Alexander:** conceptualization, methodology, writing – review and editing. **Nicholas Hirst:** conceptualization, methodology, writing – review and editing. **Neil Pillinger:** conceptualization, methodology, writing – review and editing. **Linda Denehy:** conceptualization, methodology, writing – review and editing. **Bernhard Riedel:** conceptualization, methodology, writing – review and editing. **Chelsia Gillis:** conceptualization, methodology, writing – review and editing. **Sharon Carey:** conceptualization, methodology, writing – review and editing. **Kate McBride:** conceptualization, methodology, writing – review and editing. **Kate White:** conceptualization, methodology, writing – review and editing. **Haryana M. Dhillon:** conceptualization, methodology, writing – review and editing. **Patrick Campbell:** conceptualization, methodology, writing – review and editing. **Raaj Kishore Biswas:** conceptualization, methodology, writing – review and editing. **Daniel Steffens:** conceptualization, methodology, writing – review and editing.

## Funding

The authors have nothing to report.

## Conflicts of Interest

The authors declare no conflicts of interest.

## Supporting information


Table S1

## Data Availability

Data sharing not applicable to this article as no datasets were generated or analyzed during the current study.
