# Supplementary material for: Mapping of Preoperative Screening Tools Reveals Urgent Need for Standardization in Gastrointestinal Cancer Surgery: A Scoping Review
Source: World J Surg. 2026 Mar 10;50(4):1017–48. doi: 10.1002/wjs.70313 (PMC13070451; doi:10.1002/wjs.70313)
Supplement: Supplementary file 1 — Table S1 [file WJS-50-1017-s001.docx]

## Supplementary Document 1

## Full search strategy for electronic databases.

Ovid MEDLINE(R) ALL <1946 to May 22, 2025>

| **Search string** | **Search terms** | **Results** |
| --- | --- | --- |
| 1 | exp "Surveys and Questionnaires"/ | 1313856 |
| 2 | Patient Survey$.mp. | 3841 |
| 3 | Screen$ Tool$.mp. | 36470 |
| 4 | 1 or 2 or 3 | 1342131 |
| 5 | exp Preoperative Period/ | 10253 |
| 6 | pre?operat$.mp. | 442288 |
| 7 | before surgery.mp. | 50898 |
| 8 | before operation.mp. | 9961 |
| 9 | 5 or 6 or 7 or 8 | 480713 |
| 10 | exp Neoplasms/ | 4112169 |
| 11 | neoplasm$.mp. | 3509815 |
| 12 | cancer.mp. | 2472595 |
| 13 | exp Gastrointestinal Neoplasms/ | 472564 |
| 14 | exp Colorectal Neoplasms/ | 256137 |
| 15 | exp Digestive System Surgical Procedures/ | 439748 |
| 16 | exp Colorectal Surgery/ | 4792 |
| 17 | tumo?r.mp. | 2379141 |
| 18 | malignan$.mp. | 768339 |
| 19 | 10 or 11 or 12 or 13 or 14 or 15 or 16 or 17 or 18 | 5845871 |
| 20 | exp Diet/ | 354077 |
| 21 | diet$.mp. | 970837 |
| 22 | nutrition$.mp. | 529324 |
| 23 | exp Nutrition Assessment/ | 19356 |
| 24 | exp Nutritional Support/ | 50993 |
| 25 | exp Nutrition Surveys/ | 40165 |
| 26 | nutrition$ support$.mp. | 19255 |
| 27 | exp Malnutrition/ | 141475 |
| 28 | exp Food/ | 1572540 |
| 29 | malnutrition$.mp. | 68808 |
| 30 | exp "Diet, Food, and Nutrition"/ | 2094251 |
| 31 | 20 or 21 or 22 or 23 or 24 or 25 or 26 or 27 or 28 or 29 or 30 | 2763199 |
| 32 | exp Exercise/ | 271952 |
| 33 | exercise$.mp. | 512950 |
| 34 | exp Exercise Therapy/ | 70881 |
| 35 | exp Physical Fitness/ | 39067 |
| 36 | physical fitnes$.mp. | 38505 |
| 37 | exercis$ therap$.mp. | 57816 |
| 38 | physical acitivit$.mp. | 9 |
| 39 | exp Preoperative Exercise/ | 796 |
| 40 | pre?operat$ exercise.mp. | 1115 |
| 41 | exercise train$.mp. | 23817 |
| 42 | exp Physical Therapy Modalities/ | 193195 |
| 43 | physical therap$.mp. | 71982 |
| 44 | exp Physical Fitness/ | 39067 |
| 45 | 32 or 33 or 34 or 35 or 36 or 37 or 38 or 39 or 40 or 41 or 42 or 43 or 44 | 731608 |
| 46 | exp Relaxation/ | 22662 |
| 47 | relaxation.mp. | 153257 |
| 48 | exp Cognitive Behavioral Therapy/ | 41159 |
| 49 | cognitive behavio?ral therap$.mp. | 43013 |
| 50 | exp Mindfulness/ | 8298 |
| 51 | mindfulness.mp. | 17211 |
| 52 | exp Adaptation, Psychological/ | 146213 |
| 53 | coping.mp. | 82766 |
| 54 | exp Psychosocial Intervention/ | 1463 |
| 55 | psychosocial intervention$.mp. | 9658 |
| 56 | exp Psychotherapy/ | 231727 |
| 57 | psychotherap$.mp. | 106258 |
| 58 | exp Mental Health/ | 72956 |
| 59 | psychological.mp. | 730751 |
| 60 | Psychoeducation.mp. | 5594 |
| 61 | 46 or 47 or 48 or 49 or 50 or 51 or 52 or 53 or 54 or 55 or 56 or 57 or 58 or 59 or 60 | 1201325 |
| 62 | 31 or 45 or 61 | 4490731 |
| 63 | 4 and 9 and 19 and 62 | 941 |
| 64 | limit 63 to (english language and humans and yr="2000 -Current" and "all adult") | 829 |
